# Transcriptome Analysis Reveals a Diverse Range of Novel Viruses in Australian Sugarcane Soldier Fly (*Inopus flavus*) Larvae

**DOI:** 10.3390/v16040516

**Published:** 2024-03-27

**Authors:** Gayatri Divekar, Agathe M. G. Colmant, Michael J. Furlong, Kayvan Etebari

**Affiliations:** 1 School of Agriculture and Food Sustainability, The University of Queensland, Gatton 4343, Australia; 2 Unité des Virus Émergents (UVE: Aix-Marseille Univ, Università di Corsica, IRD 190, Inserm 1207, IRBA), 13005 Marseille, France; 3 School of the Environment, The University of Queensland, Brisbane 4072, Australia

**Keywords:** soldier fly virome, transcriptome analysis, next-generation sequencing, insect-specific viruses

## Abstract

In Australia, Soldier flies (*Inopus* spp.) are economically significant pests of sugarcane that currently lack a viable management strategy. Despite various research efforts, the mechanisms underlying the damage caused by soldier fly larvae remain poorly understood. Our study aims to explore whether this damage is associated with the transmission of plant viruses during larval feeding. We also explore the larval transcriptome to identify any entomopathogenic viruses with the potential to be used as biocontrol agents in future pest management programs. Seven novel virus sequences are identified and characterised using de novo assembly of RNA-Seq data obtained from salivary glands of larvae. The novel virus sequences belong to different virus families and are tentatively named SF-associated anphevirus (SFaAV), SF-associated orthomyxo-like virus (SFaOV), SF-associated narna-like virus (SFaNV), SF-associated partiti-like virus (SFaPV), SF-associated toti-like virus (SFaTV-1 and SFaTV-2) and SF-associated densovirus (SFaDV). These newly identified viruses are more likely insect-associated viruses, as phylogenetic analyses show that they cluster with other insect-specific viruses. Small RNA analysis indicates prominent peaks at both 21 nt and 26–29 nt, suggesting the activation of host siRNA and piwiRNA pathways. Our study helps to improve understanding of the virome of soldier flies and could identify insect viruses for deployment in novel pest management strategies.

## 1. Introduction

Australia exports more than AUD 1.5 billion of sugar annually, making it the third largest exporter of this commodity globally. Sugarcane is vulnerable to insect pests and diseases and its susceptibility is increased by the extended growth time required for crops to reach maturity. Canegrubs (Order: Coleoptera) and soldier flies (Order: Diptera) are major insect pests, and they cause significant yield losses in some sugarcane regions in Australia. Soldier flies represent a species complex that comprises at least six endemic species that are economically important pests of sugarcane [1]. The most well-studied species, *Inopus rubriceps* Macquart (Diptera: Stratiomyidae) extends throughout eastern Queensland and New South Wales and populations have established in New Zealand and California, USA [2]. The focus of this study is on *I. flavus* (James) which is known to have a limited and localised distribution in eastern central Queensland, [3] but the damage that they cause has become more obvious in recent years [4]. Little is known about more recently discovered species and their distributions; the damage that they cause and their susceptibility to pathogens needs to be understood [5]. Managing soldier fly pests in sugarcane crops is challenging due to their cryptic feeding behaviour, the ineffectiveness of insecticides; the lack of access to tolerant sugarcane varieties adds another layer of difficulty for pest management. To develop improved pest management strategies, a better understanding of the relationship between soldier flies and their natural enemies is required and this could be the basis for novel future control strategies.

Generally, our knowledge of the prevalence and biodiversity of insect-specific viruses in insect populations is very limited; this is particularly the case for those viruses that infect agricultural pests. To date, most research on viruses for insect pest control has focused on baculoviruses (arthropod-specific DNA viruses) [6,7] and the benefits offered by other viruses in this context has received little attention or recognition. When deployed as biological control agents, insect-specific RNA viruses are capable of causing significant reductions in the field populations of agricultural and forestry pests [8] and aerial applications of tetraviruses and picorna-like viruses have been especially successful against leaf-eating caterpillar pests in oil palm and coconut tree plantations [9,10]. There is also a growing body of evidence which shows the potential use of insect-specific RNA viruses to genetically engineer crops for pest control [8,11]. For example, transgenic plants expressing insect picorna-like viruses can be generated and tomato plants engineered with the Norwalk virus capsid protein (NVCP) to assemble virus-like particles (VLPs) [12].

The advent of next-generation sequencing (NGS) technology has created a great opportunity for novel virus discovery, and it enables investigations into their biodiversity. These novel viruses could be insect pathogens that kill their hosts or affect their performance and development through sub-lethal effects. Other viruses might be plant pathogens which use insects as vectors and are transferred during feeding. In either case, the viruses are worthy of investigation, to determine if they damage crops or if they have the potential for development as biological control agents.

In this study, we used total RNA sequencing to investigate the virome of soldier fly larvae. Previously, employing this approach, we described the identification of a novel dicistro-like virus [13] and a novel jingmenvirus [14] in our RNA-Seq data. Subsequently, we expanded our analysis to further explore the presence of additional viral sequences. In our current study, the evidence of persistent infection for some of these viruses was provided by small RNA read profiling and it allowed us to investigate the small RNA responses across these diverse virus families. This study sheds light on the diversity of viruses present in soldier fly salivary glands. Further investigation of the impact of these newly identified viruses on soldier fly populations in different regions will enhance our understanding of the potential interactions between insect-specific viruses and their hosts. Such insights could potentially lead to the identification of new biological control agents for one of the most significant pests of sugarcane.

## 2. Material and Methods

### 2.1. Sample Collection and RNA Extraction

Sugarcane yellow soldier fly (*Inopus flavus*) larvae were collected from an infested sugarcane field near Hay Point, Queensland (21°18′5″ S, 149°14′7″ E). Sugarcane stools were excavated from the ground and large larvae were manually collected from the roots and associated soil. Larvae were transported to the University of Queensland’s laboratory for viral discovery based on next-generation sequencing. This is considered an unbiased approach, as no attempt was made to enrich viral particles through filtration, centrifugation or nuclease treatment. Total RNA samples were extracted from the salivary glands of root-exposed and starved larvae as previously described in Etebari et al., 2020 [4]. Briefly, the larval body surfaces were disinfected by soaking in 75% ethanol for 30 s and rinsed in phosphate-buffered saline (PBS) before dissecting out the salivary glands. The salivary glands (SG) were extracted by pulling out the head capsule and removing all other tissues, such as fat body droplets. The SG tissue from 20 larvae (representing one biological replicate) were pooled together and transferred to Qiazol lysis reagent for RNA extraction according to the manufacturer’s instruction (QIAGEN; Cat No.: 79306). After DNase treatment and checking the RNA quality, total RNA from six samples (three biological replicates for root-exposed larvae, three biological replicates for starved larvae) were submitted to the Australian Genome Research Facility (AGRF) for next-generation RNA sequencing. The PCR-based cDNA libraries were prepared using the Illumina TrueSeq cDNA library construction kit. cDNA from both sets of samples were sequenced using Illumina HiSeq 4000 paired read (75 × 75 bp) technologies with an average fragment size of 350 bp and insert size of 230 bp. Deep sequencing raw data have been deposited in the National Centre for Biotechnology Information’s (NCBI’s) Gene Expression Omnibus (GEO) and are accessible through GEO series accession number GSE127658.

### 2.2. Transcriptome DATA Analysis and Virus Discovery

In this study, the CLC Genomics Workbench version 20.0.1 (Qiagen, Hilden, Germany) was used for bioinformatics analyses. All libraries were trimmed from any remaining vector or adapter sequences. Low-quality reads (quality score below 0.05) and reads with more than two ambiguous nucleotides were discarded. All reads were mapped to black soldier fly, *Hermetia illucens*, as the proxy genome reference (GCF 905115235.1) to remove host-derived reads, and unmapped reads were retained for de novo assembly and virus discovery. The contigs were constructed with kmer size 45, bubble size 50, and a minimum length of 500 bp, then corrected by mapping all reads against the assembled sequences (minimum length fraction = 0.9, maximum mismatches = 2). The generated contigs were compared to the NCBI viral database using local BLAST and BLASTx algorithms. The e-value was set to 1 × 10^−10^ to maintain high sensitivity and a low false-positive rate. To detect highly divergent viruses, domain-based searches were performed by comparing the assembled contigs against the Conserved Domain Database (CDD) version 3.14 [15] and Pfam v32 [16] with an expected value threshold of 1 × 10^−3^. Sequences with positive hits to virus polymerase (RNA-dependent RNA polymerase (RdRp) domain: cd01699) were retained and further checked against a non-redundant (nr) protein database. Contig sequences with a high degree of similarity to viral proteins were then checked for complete open reading frames (ORFs). ORFs with a minimum length of 150 aa were detected in NCBI’s ORFfinder (accessed in January 2024) [17] by using standard genetic code. Putative virus sequences were re-mapped to RNA-Seq data to inspect for sufficient coverage and possible mis-assembly. The CLC Genomic Workbench’s RNA-Seq function (min. length fraction = 0.9, max. mismatches = 2, insertion cost = 3, deletion cost = 3) on a non-strand-specific option was used. TPM (Transcripts Per Kilobase Million) and Trimmed Mean of M values (TMM) normalisation was used to apply effective library sizes. As the full genome of the host is not available at the moment, we did not include Endogenous Viral Elements (EVEs) in this study.

### 2.3. Phylogenetic Analysis

The deduced amino acid sequence of predicted ORF regions of newly identified viruses were used to estimate their phylogenetic relationship with other respective members of each family. Closely related viruses from BLASTp analysis of the NCBI non-redundant protein database were downloaded. Multiple amino acid sequence alignments with relevant reference sequences were performed with the multiple alignment tool MAFFT (version 7) [18]. The maximum likelihood phylogenetic trees were inferred in IQ-TREE (version 2.2.2.6 released in May 2023) [19] using a JTT substitution matrix and assuming a discretised gamma rate distribution with four rate categories and with 1000 bootstraps in the ultrafast bootstrap analysis parameter. An appropriate outlier group was selected for each tree.

### 2.4. Viral Derived Small RNA Analysis

For analysis of the host RNAi response to identified novel viruses, a small RNA library was generated from one of the pools of 20 individuals (starved) using the NEBNext^®^ Multiplex Small RNA Library Prep Kit for Illumina^®^ at the Novogene Genomics Singapore Pte Ltd. The purified cDNA libraries were sequenced on a Novaseq 6000 (SE50), and raw sequencing reads were obtained using Illumina’s Sequencing Control Studio software. Raw data were stripped of adapters, and reads with a quality score above 0.05 and fewer than two ambiguous nucleotides were retained. Reads without 3′ adapters and also reads with fewer than 16 nt were discarded. The clean reads were mapped to each of the recently identified viruses. We examined both the size distribution of the viral-derived RNA fragments as well as “hot-spot” genomic locations for each identified virus.

## 3. Results and Discussion

We prepared the RNA-Seq libraries of six pools of salivary gland tissues from 20 soldier fly larvae per pool, collected from north Queensland, Australia. Three RNA-Seq libraries were sequenced from specimens under starvation stress and three libraries corresponded to specimens fed by sugarcane roots. Our data show that starvation stress did not meaningfully change the number of viral-derived reads in soldier fly larvae (Table 1). Overall, between 44% and 58% of total RNA reads mapped to identified viral sequences. With these reads, we identified several RNA virus sequences from the *Narnaviridae*, *Totiviridae*, *Partitiviridae*, *Orthomyxoviridae* and *Xinmoviridae* families in soldier fly salivary glands. We also identified viral sequences from the *Dicistroviridae* and *the currently unclassified flavi-like jingmenvirus genus* in our datasets (Table 1), but these have been reported previously [13,14]. In addition, we found the full genome sequence of a non-enveloped single-stranded DNA virus from the *Parvoviridae* family. Identifying a viral sequence through a metagenomic survey does not necessarily determine the host for those novel viruses. Typically, viruses found in insects through next-generation sequencing can include viruses of plants, fungi, and protozoa. We chose “soldier fly-associated viruses” to name our newly identified virus sequences due to a limitation of this approach: the challenge of accurately assigning hosts to novel virus sequences.

### 3.1. Soldier Fly-Associated Anphevirus

**Figure 1:**
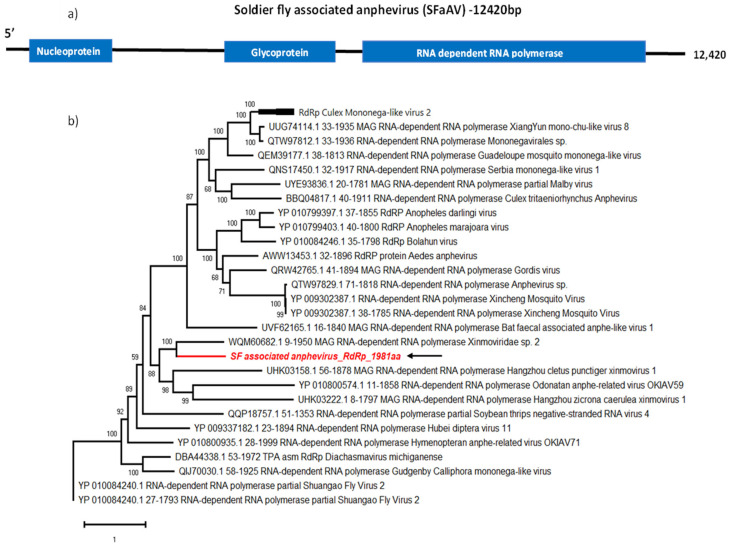
Genome organisation of SFaAV (**a**) shown with three ORFs encoding RdRp, glycoprotein and nucleoprotein. For phylogenetic analysis of RdRp (**b**), the amino acid sequence of novel SFaAV (highlighted in red and indicated with arrow) is shown along with aligned reference sequences. Maximum Likelihood tree is constructed with 1000 bootstrap replicates and JTT substitution matrix with four gamma-variable sites. The scale bar refers to the number of amino acid changes per site. Bootstrap support values >50% are shown at the nodes.

The virus family *Xinmoviridae* includes 12 genera, one of which is the *Anphevirus* genus [20,21]. Members of this family have −ssRNA genomes of approximately 12 kb in length and their structure has yet to be elucidated [22]. The only member species listed according to the International Committee on Taxonomy of Viruses (ICTV) report is the Xincheng mosquito virus (*Anphevirus xinchengense*) [20]. *Xinmoviridae* family members are known to have arthropod hosts and anpheviruses have been isolated worldwide from mosquitoes [22,23,24]. In this study, we discovered a novel anphevirus sequence from soldier fly larvae salivary glands, and tentatively named it Soldier fly-associated anphevirus (SFaAV). The predicted SFaAV genome encodes for a 1981 amino acid-long RNA-dependent RNA polymerase. It also encodes for a 643 aa glycoprotein and a 438 aa nucleoprotein (Figure 1a).

We used BLASTp to determine the most closely related previously reported virus sequences. The most similar sequence (40.6% identity) was from an unclassified virus named Medvezhye Haematopota Xinmo-like virus (WQM60682.1), detected in *Haematopota pluvialis* flies (Table 2).

The phylogenetic analysis of SFaAV and other anphevirus RdRp sequences groups SFaAV with other insect-associated anpheviruses, including the sequence identified with BLASTp, as well as Hangzhou cletus punctiger xinmovirus 1 (UHK03158.1) detected from *Cletus punctiger* (Hemiptera: Coreidae), Odonatan anphe-related virus OKIAV59 (YP010800574.1) detected in *Cordulegaster boltonii* (Odonta: Cordulegastridae) and Hangzhou zicrona caerulea xinmovirus 1 (UHK03222.1) detected in *Zicrona caerulea* (Hemiptera: Pentatomidae) (Figure 1b).

### 3.2. Soldier Fly-Associated Orthomyxo-like Virus

The most common species of the *Orthomyxoviridae* family are the influenza viruses containing four genera: *Alphainfluenzavirus, Betainfluenzavirus*, *Gammainfluenzavirus* and *Deltainfluenzavirus*. Aside from these, the family includes the genera, *Mykissvirus*, *Sardinovirus*, *Isavirus*, *Thogotovirus* and *Quaranjavirus*. While the influenza viruses have primarily human hosts, thogotoviruses and quaranjaviruses have arthropod hosts [25,26]. Orthomyxoviruses have a segmented −ssRNA genome which usually contains 6–8 segments, each encoding a different protein [27].

**Figure 2:**
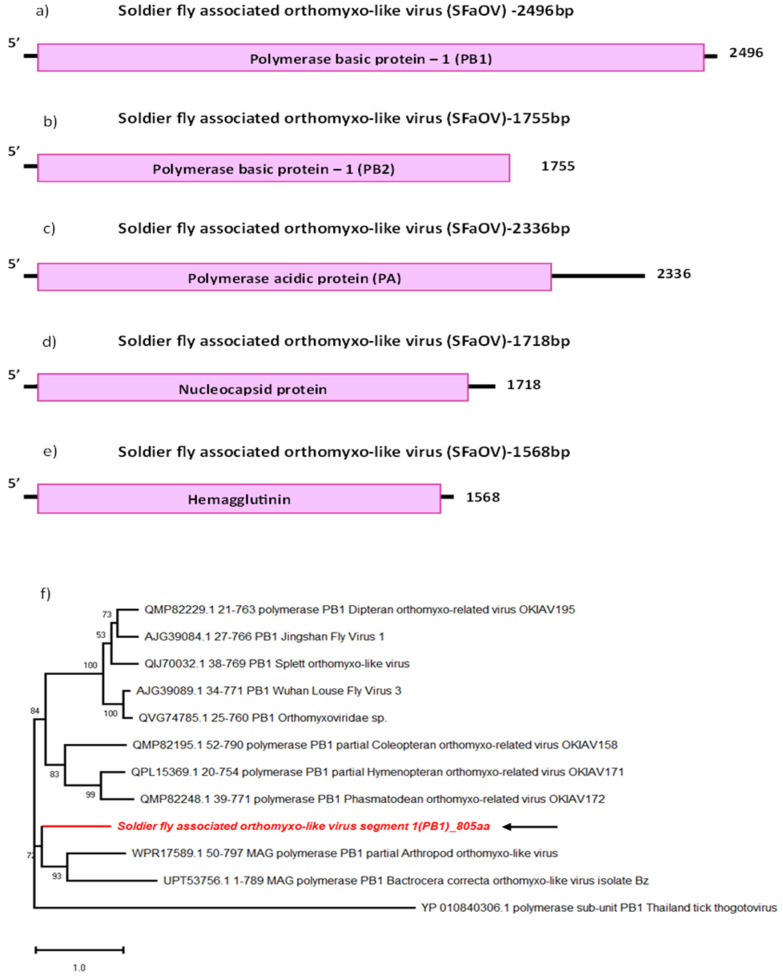
Genome organisation of all segments of SFaOV: PB1 (**a**), PB2 (**b**), PA (**c**), Nucleocapsid (**d**) and Hemagglutinin (**e**). For phylogenetic analysis (**f**), the amino acid sequence of PB1 segment of SFaOV (highlighted in red and indicated with arrow) is shown along with aligned reference sequences. Maximum Likelihood tree is constructed with 1000 bootstrap replicates and JTT substitution matrix with four gamma-variable sites. The scale bar refers to the number of amino acid changes per site. Bootstrap support values >50% are shown at the nodes. ML trees of remaining segments of SFaOV are given in Supplementary File S1.

We were able to identify five segments of a novel orthomyxo-like virus sequence, tentatively named Soldier fly-associated orthomyxo-like virus (SFaOV) (Figure 2b–e). The sequences identified correspond to three segments coding for the peptides composing the RdRp: polymerase basic segment 1 (PB1), polymerase basic segment 2 (PB2) and polymerase acidic segment (PA), as well as a nucleocapsid (NC) and a hemagglutinin segment (HA). Each of these segments was found to encode a single protein. With the exception of the PA segment, which had a theoretical isoelectric point of 5.31, all the other segments had a basic pI of >9, suggesting differences in the transport activity of the proteins produced by each segment. All segments show sequence similarity with unclassified members of the *Orthomyxoviridae*. The most closely related sequences to the SFaOV segments are sequences from Arthropod orthomyxo-like virus (WPR17589.1), detected in *Oribatida* mites from New Zealand (44.39% and 28.27% identity respectively), according to the BLASTp results and phylogenetic analysis (Table 2 and Figure 2f). SFaOV segment sequences also cluster with Soybean thrips quaranja-like virus 1 (QPZ88432.1), Bactrocera correcta orthomyxo-like virus isolate Bl (UPT53725.1) and Bactrocera tryoni orthomyxo-like virus (UPT53749.1) detected in *Bactrocera* fruit flies as well as Coleopteran orthomyxo-related virus OKIAV196 (QMP82407.1), all detected in arthropods (Table 2 and Figure 2f) [28].

The average read coverage for the nucleocapsid protein segment of this virus was around 102, which is higher than other segments (Table 1). The small RNA read coverage graphs also indicated notably low coverage for the virus sequences, and was particularly noticeable in the PB2 segment (Supplementary File S1). This suggests the possibility that, despite the clustering of this novel orthomyxo-like sequences with other arthropod-associated virus sequences, there may not be active replication of this virus in the salivary glands. Further investigation is necessary to explore tissue tropism for this novel virus in soldier fly larvae and to determine its ability to infect and replicate within specific cell types or tissues in this species.

### 3.3. Soldier Fly-Associated Narna-like Virus

Viruses belonging to the *Narnaviridae* family have a +ssRNA genome with a single ORF encoding for a single large protein, the RdRp. Their genome is around 3 kb long and is ’naked’, meaning the virus has no viral envelope or capsid [29,30]. Typically, narnaviruses are associated to fungal hosts, but recently they have also been discovered in insects. Indeed, a recent study reported the detection of a narnavirus replicating in a *Culex tarsalis* cell line, free from any fungal or bacterial contamination, by small RNA sequencing [31]. Another narnavirus has been detected in *Aedes japonicus* mosquitoes, by metagenomics [23]. Recent articles report the detection of narnavirus sequences in *Forcipomyia taiwana* (Diptera: Ceratopogonidae) biting midges [32], horse flies (Diptera: Tabanidae) [33] and parasitoid wasps (Order: Hymenoptera) [34].

**Figure 3:**
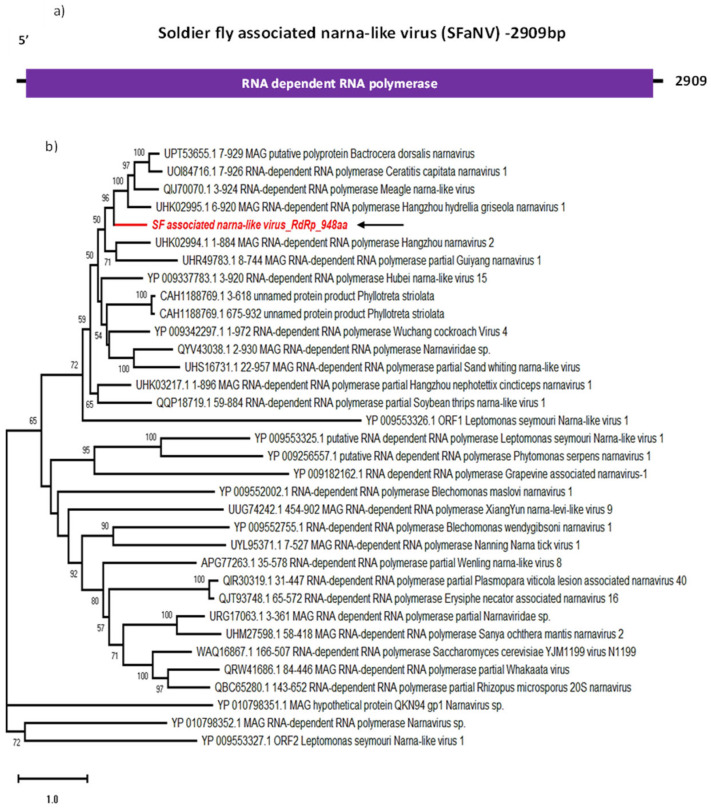
Genome organisation of SFaNV (**a**) showing single ORF encoding RdRp. For phylogenetic analysis of RdRp (**b**), the amino acid sequence of SFaNV (highlighted in red and indicated with arrow) is shown along with aligned reference sequences. Maximum Likelihood tree is constructed with 1000 bootstrap replicates and JTT substitution matrix with four gamma-variable sites. The scale bar refers to the number of amino acid changes per site. Bootstrap support values >50% are shown at the nodes.

Here, we report the identification of an SF-associated narna-like virus (SFaNV) 2909 bp long genome sequence, with a single ORF encoding for a 948 aa RdRp with a 106.3 kDa molecular weight (Figure 3a). The most closely related sequences to SFaNV are the RdRp of Hangzhou hydrellia griseola narnavirus 1 (UHK02995) (Table 2), Bactrocera dorsalis narnavirus (UPT53655) and Meagle narna-like virus (QIJ70070), with over 48% identity according to BLASTp. These three virus sequences were discovered from insect-derived samples [28] and cluster with SFaNV by phylogenetic analysis as well (Figure 3b).

The number of RNA-Seq reads mapped to this viral sequence varies between 157 and 428, with an average assembly coverage of 6.98 (Table 1). This suggests that the newly identified virus is less prevalent across all RNA libraries as compared to the total and small RNA read coverage of the previously found jingmenvirus and dicistro-like virus from the same dataset. This indicates the presence of fewer copies of the SFaNV genome in the salivary glands of soldier fly. Since exact tissue tropism of narnavirus in insects is unknown, it can be assumed that salivary glands may not be the site of active replication. Indeed, larvae may have ingested fungus carrying SFaNV; in that case, any ingested virus would primarily be found in the midgut rather than the salivary glands of the insect. Further research is required to elucidate the role of SFaNV in soldier flies.

### 3.4. Soldier Fly-Associated Partiti-like Virus

*Partitiviridae* have a bipartite 3–5 kb long dsRNA genome, with each genomic segment encapsidated separately. One segment encodes the RdRp, with a reverse transcriptase domain, while the other encodes the coat protein [35,36]. Similarly to narnaviruses, they are known to primarily infect fungi and plants. Recent studies show that partitiviruses can also replicate in arthropods [37,38], and even be transmitted vertically from parent to progeny *Drosophila melanogaster* or *Aedes aegypti* [39]. Reports by Xu et. al., (2020 and 2022) have demonstrated the lethality of partitiviruses in lepidopteran hosts [37,38]. As the name indicates, the partitivirus genome is made of bisegmented dsRNA which is separately encapsidated.

**Figure 4:**
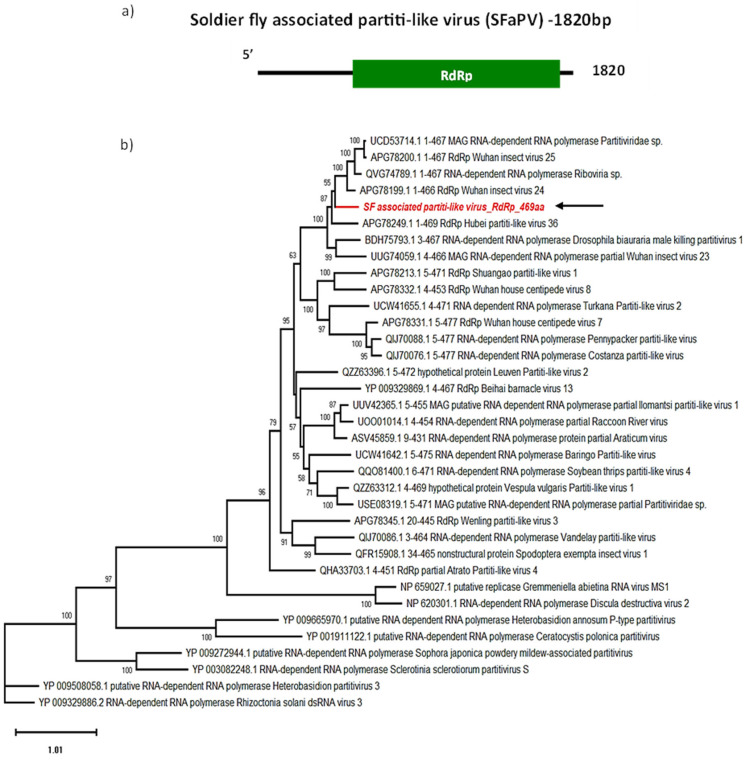
Genome organisation of SFaPV (**a**) showing single ORF encoding RdRp. For phylogenetic analysis of RdRp (**b**), the amino acid sequence of SFaPV (highlighted in red and indicated with arrow) is shown along with aligned reference sequences. Maximum Likelihood tree is constructed with 1000 bootstrap replicates and JTT substitution matrix with four gamma-variable sites. The scale bar refers to the number of amino acid changes per site. Bootstrap support >50% are shown at the nodes.

We identified the partial sequence of a novel SF-associated partiti-like virus (SFaPV), with one segment encoding a 469 aa long ORF predicted to be the RdRp, with a 53.75 kDa molecular weight and an approximate isoelectric point at 9.46, including the expected reverse transcriptase domain (Figure 4a). The second segment encoding the coat protein was not identified. According to BLASTp and the phylogenetic analysis, SFaPV is most closely related (>67% identity) to insect-associated partitivirus sequences: Wuhan insect virus 24 (APG78199.1) (Table 2), unclassified *Riboviria* sp. (QVG74789.1), unclassified *Partitiviridae* sp. (UCD53714.1), Hubei partiti-like virus (APG78249.1) [40] and Jalime partitivirus (QVU40013.1) [41] (Figure 4b).

From a pest control point of view, it is important to note that a partitivirus related to SFaPV, Drosophila male-killing partitivirus, was found to encode a gene which favours female selection by elimination of males [42]. These new findings indicate that male killing by these viruses may be prevalent among insect species and could be potentially utilised to suppress host population. This avenue could be explored for SFaPV in soldier flies. In addition to the male-killing effect observed in *Drosophila*, other closely related partiti-like viruses have been reported from the African armyworm, *Spodoptera exempta* and *S. frugiperda* [37,38]. In *S. frugiperda*, these viruses had detrimental effects on larvae. Subsequently, these viruses were found to infect the Egyptian armyworm, *Spodoptera littoralis*, leading to larval and pupal mortality [38].

The average assembly coverage of SFaPV is around 7, with between 73 and 296 RNA reads mapped to this viral sequence (Table 1). This indicates that these viruses are among the less abundant SF viruses, characterised by low mapping coverage and small RAN profile (Supplementary File S1).

### 3.5. Soldier Fly-Associated Toti-like Virus

Members of the five genera of the *Totiviridae* family have encapsidated 4.6–7 kb long dsRNA genomes [43,44]. Totiviruses were traditionally associated with fungal hosts such as *Saccharomyces cerevisiae*, as Saccharomyces cerevisiae virus L-A is the type species of the Totivirus genus. In recent years, novel totiviruses have been detected in arthropods from Europe [23], Australia [45,46], Asia [47] and South America [48]. Totiviruses have also been detected in plants worldwide, notably in Australia [49], China [50] and Ecuador [51].

**Figure 5:**
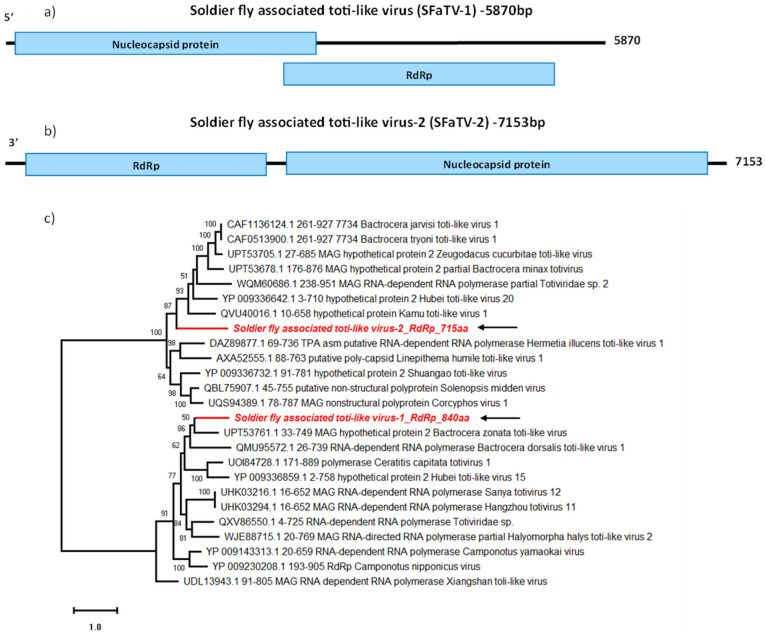
Genome organisation of SFaTV-1 (**a**) shows two overlapping ORFs encoding Nucleocapsid and RdRp on the forward strand. Genome structure of SFaTV-2 (**b**) shows non-overlapping ORFs encoding Nucleocapsid and RdRp on the reverse strand. Phylogenetic analysis of RdRp of both novel totiviruses using amino acid sequences (**c**) is shown along with aligned reference sequences. The novel totiviruses are highlighted in red and indicated with arrows. Maximum Likelihood tree is constructed with 1000 bootstrap replicates and JTT substitution matrix with four gamma-variable sites. The scale bar refers to the number of amino acid changes per site. Bootstrap support >50% are shown at the nodes. ML tree is midpoint rooted for easier visualisation.

In this study, we detected two novel toti-like virus sequences tentatively named SF-associated toti-like viruses (SFaTV). The assembled sequences for SFaTV-1 and SFaTV-2 were 5870 nt and 7153 nt long, respectively. Both SFaTV-1 and SFaTV-2 sequences contain two ORFs (Figure 5a,b); the larger ORF (SFaTV-1: 1050 aa; SFaTV-2: 1487 aa) encodes for the nucleocapsid and the smaller ORF (SFaTV-1: 840 aa; SFaTV-2: 715 aa) encodes for the RdRp. The translation of SFaTV-1 ORFs involves -1 ribosomal frameshifting as previously seen in totiviruses [43].

Based on BLASTp results, SFaTV-1 is most closely related to Bactrocera zonata toti-like virus (UPT53760.1), while SFaTV-2 is most closely related to Zeugodacus cucurbitae toti-like virus (UPT53705.1), both detected in fruit flies (Table 2). Interestingly, while both sequences are grouped with insect-associated totivirus sequences, they did not cluster together in the phylogenetic analysis, despite originating from the same samples (Figure 5c).

### 3.6. Soldier Fly-Associated Densovirus

Densoviruses are small, non-enveloped ssDNA viruses belonging to the sub-family Densovirinae of family *Parvoviridae* which contains 11 genera [52,53]. Mosquito densoviruses belong to the *Brevidensovirus* and *Ambidensovirus* genera of the Densovirinae sub-family and are known to exclusively infect invertebrates and to have the potential to be lethal when actively replicating in the midgut of their mosquito hosts [54,55]. Mosquito densoviruses have previously been used as a mosquito population management tool [56,57], including in combination with *Bacillus thuringenesis* toxins [58] and as a larvicidal [59]. These strategies could be investigated to manage soldier fly larvae populations, as we have detected a densovirus sequence in our soldier fly larvae-derived samples, named Soldier fly-associated densovirus (SFaDV).

**Figure 6:**
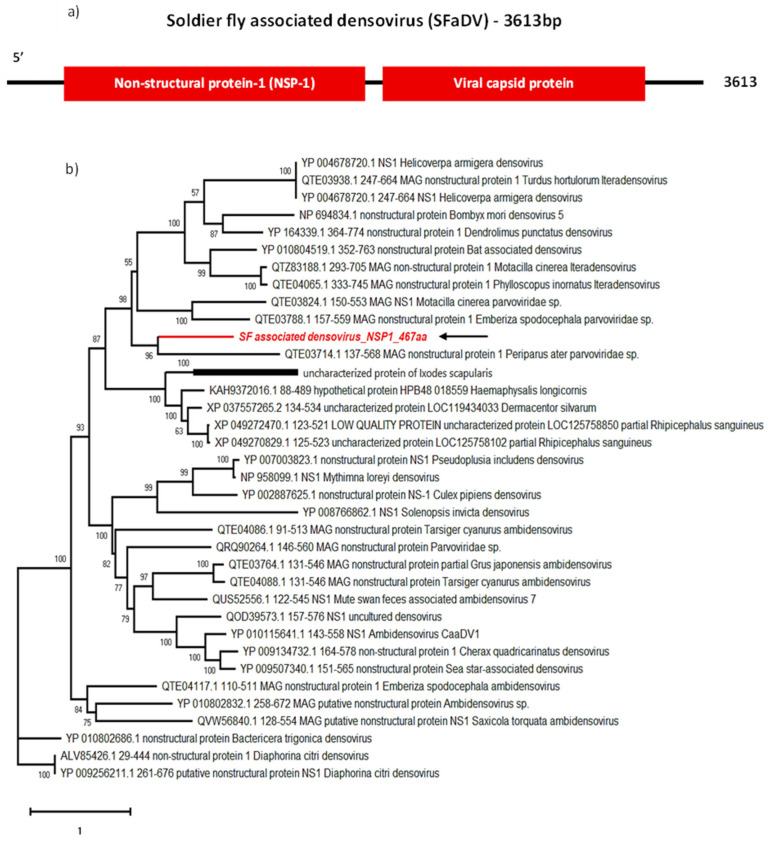
Genome organisation of SFaDV (**a**) showing two ORFs encoding NSP-1 and viral capsid protein. For phylogenetic analysis of NS1 (**b**), the amino acid sequence of SFaDV (highlighted in red and indicated with arrow) is shown along with aligned reference sequences. Maximum Likelihood tree is constructed with 1000 bootstrap replicates and JTT substitution matrix with four gamma-variable sites. The scale bar refers to the number of amino acid changes per site. Bootstrap support values >50% are shown at the nodes.

The 3616 nt long genome we obtained contained two ORFs (Figure 6a): ORF1 coding for a 467 aa long non-structural protein (NS1, molecular weight 54.0 kDa and pI 9.23) and ORF2 coding for a 358 aa long capsid protein (VP1). SFaDV NS1 contains the conserved helicase of superfamily 3 domain, common to all *Parvoviridae*, and VP1 contains a phospholipase A2-like domain often found on the N-terminal region of Parvovirus VP1 [60,61]. These findings satisfy the demarcation criteria to be included in the *Parvoviridae* family: having a large coding region of a non-structural (NS1) protein containing an SF3 helicase domain along with the coding region of a viral capsid (VP) protein [52].

**Figure 7:**
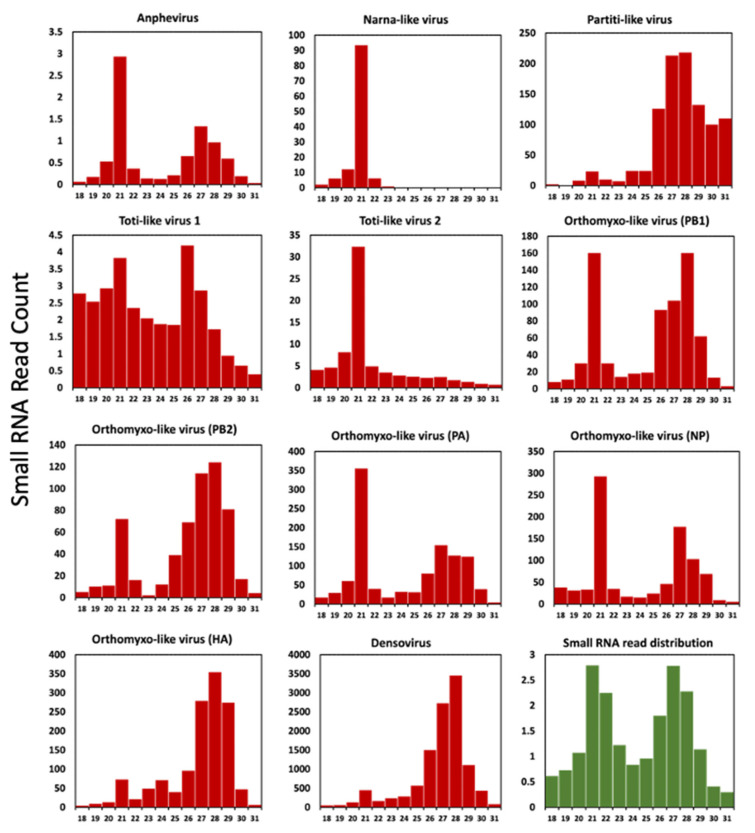
Virus-derived small interfering RNA profile of recently identified solider fly-associated viruses. Read count on *Y*-axis is in thousands. The chart with green bars represents total small RNA read distribution, read count on *Y*-axis is in millions.

In addition, according to BLASTp, the closest relatives of SFaDV are the two iteradensoviruses: Motacilla cinerea iteradensovirus (QTZ83188.1) detected in birds, and Helicoverpa armigera densovirus (HaDV) detected in Lepidoptera (YP_004678721.1), depending on the ORF (Table 2). Phylogenetic analyses of NS1 and VP1 cluster SFaDV with parvoviruses detected in birds, such as *Periparus ater Parvoviridae* sp. (PaPV) (QTE03714.1) (Figure 7). Identifying insect-specific viruses in insectivorous birds is a common occurrence. Despite being isolated from the cloaca of birds, Motacilla cinerea iteradensovirus (QTZ83188.1) and PaPV are more likely to be insect viruses. It has been hypothesised that these birds potentially feed on infected fruit flies (*Drosophila erecta*), indicating that they are not likely avian viruses [62]. However, further investigations are necessary to confirm host specificity for this newly identified SFaDV.

### 3.7. Virus-Derived Small RNA Profile

To generate a small RNA profile and analyse the length and position distributions of small RNAs in the viral genome, a small RNA library was constructed from a pool of 20 larvae. These larvae had previously undergone Total RNA-Seq analysis for virus discovery. We explored the virus-derived small interfering RNAs (vsiRNAs) profile for all solider fly new virus sequences (Figure 7 and Supplementary File S1). We retained the small RNA reads from 18–31 nt after trimming the adapters, and size distributions were generated.

The small interfering RNA of around 21 nt is created by the cleavage of viral RNA by the RNase-III endonuclease Dicer-2. An RNA-induced silencing complex (RISC) is formed by loading of the cleaved siRNA onto Argonaute-2 protein [63]. These vsiRNAs are loaded into the RISC target RNA molecules through complementarity, reducing virus gene transcription and ultimately virus replication. This mechanism has previously been described for many insect RNAs [23,64,65] and DNA viruses [4]. For most insect viruses, the vsiRNAs display a sharp peak at 21 nt and are symmetrically distributed throughout the viral genome [28,66]. This signifies a strong antiviral response by the host against all regions of the viral genome, which in turn indicates active replication of virus in the insect host [23].

The length distribution of the viral small RNA profile created by the soldier fly RNAi pathway showed a prominent peak at 21 nt (proportionally higher compared to read count at other lengths for the same virus) in SFaAV and SFaNV (Figure 7). Based on this, it can be theorised that these viruses are actively replicating, thus triggering the host siRNA pathway. The virus-derived small RNA profile for all segments of SFaOV (except the HA segment) showed peaks at both 21 nt and the 26–29 nt range (Figure 7). Peaks at 26–29 nt indicate the activity of piwi-interacting RNAs (piRNAs). Orthomyxoviruses are known to elicit both siRNA and piRNA antiviral responses [28,67]. It is possible that the required recognition and conformational changes for siRNA pathway activation did not occur in the SFaOV hemagglutinin segment. This could be due to the low and uneven distribution of the HA segment (Supplementary File S1), which needs to be investigated further. Generally, piRNAs cleave some viruses in insect hosts, although their main function is silencing of transposons using the ping-pong pathway [68]. These piRNAs are primarily derived from transposable elements (TEs). Given that the novel viruses exhibit piRNA activity, it is possible that their genomes may complement those of the TEs that generate these piRNAs. A report by Nigg et al., (2020) [68] shows EVEs derived from Diaphorina citri densovirus (DcDV) produce piRNA which specifically target DcDV, a DNA virus, and no other naturally infecting RNA viruses. However, exclusive piRNAs activity is observed in both SFaDV (a DNA virus) and SFaPV (an RNA virus); this occurrence of EVE-derived piRNA can also be studied for soldier fly viruses, especially for SFaPV, which shows very low small RNA reads coverage (Supplementary File S1) and a partial genome.

Interestingly, the pattern of small RNA read distribution for the two newly identified totiviruses differs. SFaTV-1 does not display the typical RNAi response profile with a peak at 21 nt, unlike SFaTV-2, where RNA silencing is likely active (Figure 7). However, the pattern of small RNA reads mapping to the entire viral genome in both of these totiviruses does not exhibit a symmetric pattern. For example, more reads have been mapped to the positive strand of SFaTV-1 and to the negative strand for SFaTV-2 (see Supplementary File S1). The majority of total RNA reads used to assemble SFaTV-1 are derived from only one library (Table 1), suggesting that this virus may not be highly prevalent in the population, and the RNAi pathway may not be active against it. Additionally, it can be speculated that there is piRNA activity against SFaTV-1, similar to that observed in the other dsRNA virus, SFaPV. It has been previously reported that piRNA exhibits a coding strand bias [69], which may account for the 25–27 nt peaks observed only in SFaTV-1 and not SFaTV-2. The total assembly coverage for SFaTV-1 and SFaTV-2 is 10.69 and 15.11, respectively. High read counts were recorded for SFaTV-2 in all control libraries (ranging from 1353 to 3725 reads), while the total read count for samples subjected to starvation stress noticeably decreased to a range of 403 to 776 reads (see Table 1). All these differences in their small RNA read profiles can be correlated with the fact that they do not cluster together in the phylogenetic tree. The distinct small RNA profiles observed for SFaTV-1 and SFaTV-2, as well as their differing mapping patterns and phylogenetic relationships, emphasise the necessity for further investigation to comprehend the complexity of viral interactions within the soldier fly population.

## 4. Conclusions

The *Inopus* genus has been extensively studied for its significant impact on the sugarcane industry, but the mechanism of damage remains poorly understood. We investigated the virome of larval salivary glands to identify any potential pathogenic plant viruses, as we hypothesised that this damage may be linked to the transmission of plant viruses during larval feeding. In this study, we did not identify any known pathogenic plant viruses. However, we report the discovery of seven novel virus sequences belonging to specific virus families *Xinmoviridae*, *Parvoviridae*, *Narnaviridae*, *Partitiviridae*, *Totiviridae* and *Orthomyxoviridae*. The virus-derived small RNA reads profile show peaks at both 21 nt and 26–29 nt, implying an effective host RNAi response against a variety of viruses.

The sugarcane industry has yet to identify an effective method for controlling soldier flies. However, in other agricultural sectors, viruses have been used as biocontrol agents with notable success. Baculoviruses, in particular, have been applied successfully in many agricultural settings, while there is optimism regarding the potential future use of densoviruses and partitiviruses in pest management. In this study, we were able to successfully identify viruses with a potential for use as biocontrol agents in pest management of soldier fly. Further research and continuous monitoring of the insect, plant and fungal virome can aid in better understanding of complex co-evolutionary processes between viruses and their insect hosts and help in designing effective control strategies.

## Figures and Tables

**Table 1:** Virus derived RNA read of the recently identified soldier fly-associated viruses.

Virus Name	Accession Code	Length (bp)	Average Coverage	Starved (Total Read Count)			Control (Total Read Count)		
				C1	C2	C3	SG1	SG2	SG3
SF dicistro-like virus *	MW357714	9838	387,130.38	53,083,305	61,949,103	37,296,313	52,683,814	62,908,664	43,691,654
SF jingmenvirus VP1-3 *	OM869462	2467	191,049.43	4,796,829	6,845,413	7,003,458	5,323,116	7,967,414	6,487,326
SF jingmenvirus NSP2 *	OM869461	2540	115,895.32	3,815,562	4,258,375	4,661,773	3,434,878	4,790,021	3,078,249
SF jingmenvirus NSP1seg 1 *	OM869459	2874	68,432.92	2,121,801	3,155,956	3,392,545	2,100,399	3,569,391	1,826,863
SFaAV	PP410010	12,420	26.12	4013	14,297	179	8514	264	174
SFaOV (PB1)	PP410013	2496	8.37	111	855	169	198	365	118
SFaOV (PB2)	PP410014	1755	5.92	98	209	135	185	298	79
SFaOV (polymerase PA)	PP410015	2336	5.86	176	320	172	246	360	148
SFaOV (nucleocapsid protein)	PP410016	1718	102.67	1554	2302	1988	5018	2705	936
SFaOV (hemagglutinin)	PP410017	1568	21.26	259	361	207	644	1033	306
SFaNV	PP410020	2909	6.98	428	212	157	386	280	282
SFaPV	PP410019	1820	7.07	231	115	73	288	296	184
SFaTV-1	PP410011	5870	10.69	88	540	75	946	2984	659
SFaTV-2	PP410012	7153	15.11	403	569	776	3725	2729	1353
SFaDV	PP410018	3613	40.29	415	331	402	512	11,497	448
**Viral read in the library (%)**				**51.27**	**58.00**	**46.34**	**48.32**	**51.06**	**44.41**

* These viruses were previously reported by Asselin et al. (2021) [13] and Colmant et al. (2022) [14].

**Table 2:** Similarity analysis of novel soldier fly (SF)-associated viruses with closest related viruses using BLASTp.

Name of Virus	ORF	Virus Family	Genome	Query Size (aa)	Identity (%)	Accession	Closest Hit
SFaAV	1	*Xinmoviridae*	−ssRNA	1981	40.62	WQM60682.1	RdRp [Medvezhye Haematopota Xinmo-like virus]
	3			438	26.99	WQM60679.1	ORF1 protein [Medvezhye Haematopota Xinmo-like virus]
	5			643	35.97	WQM60677.1	putative glycoprotein [Medvezhye Chrysops Xinmo-like virus]
SFaOV (PB1)	1	*Orthomyxoviridae*	−ssRNA	805	44.39	WPR17589.1	PB1 [Arthropod orthomyxo-like virus]
SFaOV (PB2)	1			567	28.05	UPT53724.1	PB2 [Bactrocera correcta orthomyxo-like virus isolate Bl]
SFaOV (PA)	1			641	28.27	WPR17586.1	PA protein [Arthropod orthomyxo-like virus]
SFaOV (Nucleocapsid)	1			516	29.4	QMP82373.1	nucleocapsid protein [Lepidopteran orthomyxo-related virus]
SFaOV (Hemagglutanin)	1			481	25.11	UPT53725.1	hemagglutinin [Bactrocera correcta orthomyxo-like virus B1]
SFaNV	1	*Narnaviridae*	+ssRNA	948	48.78	UHK02995.1	RdRp [Hangzhou hydrellia griseola narnavirus 1]
SFaPV	1	*Partitividae*	dsRNA	469	67.81	APG78199.1	RdRp [Wuhan insect virus 24]
SFaTV-1	1	*Totiviridae*	dsRNA	1050	28.49	UPT53760.1	hypothetical protein 1 [Bactrocera zonata toti-like virus]
	3			840	44.65	UPT53761.1	hypothetical protein 2 [Bactrocera zonata toti-like virus]
SFaTV-2	2	*Totiviridae*	dsRNA	715	36.77	UPT53705.1	hypothetical protein 2 [Zeugodacus cucurbitae toti-like virus]
	3			1487	25.77	YP_009333169	hypothetical protein 1 [Hubei toti-like virus 19]
SFaDV	2	*Parvoviridae*	ssDNA	467	35.55	QTZ83188.1	non-structural protein 1 [Motacilla cinerea Iteradensovirus]
	6			358	36.49	WAY26506.1	hypothetical protein [Parvoviridae sp.]

## Data Availability

The raw sequencing files are available under GEO series accession number GSE127658. Nucleotide sequence data reported is available in the GenBank database under the accession number PP410010-PP410020.

## Supplementary Materials

The following supporting information can be downloaded at: https://www.mdpi.com/article/10.3390/v16040516/s1, Supplementary file S1: The distribution of 21nt-long viral-derived sRNA mapped back to the virus positive (Blue) and negative (Red) sense nucleotide sequences.
