# Supplementary material for: Transcriptome Analysis Reveals a Diverse Range of Novel Viruses in Australian Sugarcane Soldier Fly (Inopus flavus) Larvae
Source: Viruses. 2024 Mar 27;16(4):516. doi: 10.3390/v16040516 (PMC11054854; doi:10.3390/v16040516)
Supplement: Supplementary file 1 [file viruses-16-00516-s001.zip › viruses-2933939-supplementary.pdf]

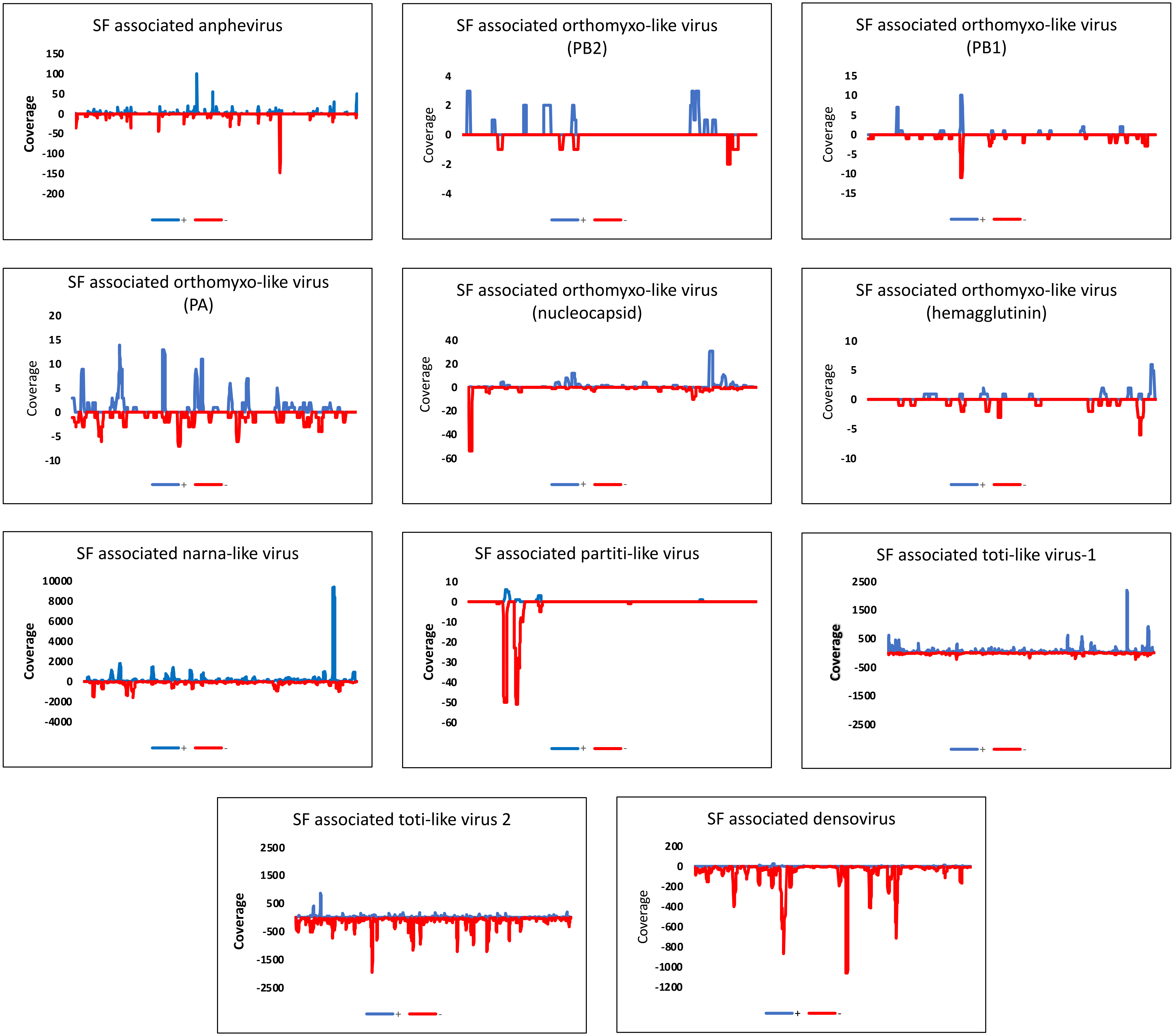

The distribution of 21nt-long viral-derived sRNA mapped back to the virus positive (Blue) and negative (Red) sense nucleotide sequences
